# A Comparative Study on the Mechanical Properties of a Polymer-Infiltrated Ceramic-Network Material Used for the Fabrication of Hybrid Abutment

**DOI:** 10.3390/ma11091681

**Published:** 2018-09-11

**Authors:** Salim Ongun, Sevcan Kurtulmus-Yilmaz, Gökçe Meriç, Mutahhar Ulusoy

**Affiliations:** 1 Department of Prosthodontics, Faculty of Dentistry, Near East University, Lefkosa, 99138 Mersin 10, Turkey; ongunsalim@gmail.com (S.O.); mutahhar.ulusoy@neu.edu.tr (M.U.); 2 Department of Prosthodontics, Faculty of Dentistry, Okan University, 34959 İstanbul, Turkey; gokcemeric@yahoo.com

**Keywords:** lithium disilicate, custom abutment, flexural strength, microshear bond strength, fracture resistance

## Abstract

Polymer-infiltrated ceramic-network (PICN) material is a new type of material used for the hybrid abutments of dental implants. This study aimed to compare flexural strength, bond strengths, and fracture-resistance values of PICN with lithium disilicate ceramic (LDS) and to evaluate the effect of thermocycling on the tested parameters. Twenty specimens were fabricated using computer-aided design and manufacturing (CAD-CAM) technology for each material according to three-point bending (*n* = 10), microshear bond strength (µSBS), and a fracture-resistance test (hybrid abutment, *n* = 10). All specimens of each test group were divided into two subgroups, thermocycled or nonthermocycled. Hybrid abutments were cemented on titanium insert bases and then fixed on implants to compare fracture resistance. Failure loads were recorded for each test and data were statistically analyzed. Thermocycling decreased bond strength to the resin luting agent and the fracture-resistance values of both materials (*p* < 0.001), whereas flexural-strength values were not affected. LDS ceramic showed significantly higher flexural strength, bond strength, and fracture-resistance values than PICN material (*p* < 0.001). Within the limitations of this study, LDS may be a preferable hybrid-abutment material to PICN in terms of mechanical and bonding properties.

## 1. Introduction

Over the past decades, esthetic has become an increasingly important factor in implant dentistry. Esthetic value has further increased especially in implants placed in the maxillary anterior region [1]. Therefore, a well-positioned implant is important for esthetic and functional success in implant-supported restorations [2].

Prefabricated titanium abutments have been used for many years in the construction of implant-supported prostheses [3]. Despite having advantages, such as accelerating gingival healing and preventing galvanism or corrosion at the abutment surface, prefabricated titanium abutments may not provide an esthetic result on the anterior region, especially in case of thin gingival phenotypes. Since the metallic color of the abutment can reflect through gingiva [4], ceramic abutments have been introduced to overcome the grayish/bluish appearance of gingival tissues, which are more successful in terms of esthetics and biocompatibility [4,5].

Prefabricated ceramic abutments are uniform, standardized, easy to use, and biocompatible. However, if the implant angle and position are not appropriate, or if the soft-tissue height is insufficient, prefabricated ceramic abutments will not provide desired esthetics [3]. In such cases, custom ceramic abutments fabricated with computer-aided design and manufacturing (CAD-CAM) systems are recommended which can imitate natural appearance since abutments are designed according to patients’ gingival contour, occlusion, and the position of the implant in the dental arch [2].

Custom abutments can be either one-piece or two-piece. One-piece abutments are completely made of ceramic material, including the implant-abutment connection. In recent years, two-piece abutments, which can also be referred to as hybrid abutments, have been developed for single-tooth implant restorations in the anterior and posterior regions. Hybrid abutments consist of a prefabricated titanium insert base (titanium bonding base), on which a customized CAD-CAM fabricated ceramic coping is cemented in the laboratory [6]. Contrary to one-piece abutment, in hybrid abutments the titanium insert is in contact with the implant platform and abutment screw rather than the ceramic coping. Therefore, it has been suggested that this design, including the titanium-to-titanium contact, reduced the risk of damage at implant-abutment interfaces [7].

Zirconia and lithium disilicate (LDS) ceramics have been used for many years as ceramic copings for hybrid abutments, and, in a recent study, both materials showed durability and strength after dynamic loading [8]. In the last few years, hybrid dental restorative materials consisting of mixed nanostructures have been developed to improve the physical and biologic behaviors of materials that are used in different fields of dentistry [9]. Polymer-infiltrated ceramic-network (PICN) material has been introduced to dental market that comprises of two interlocking components, a porous sinterized ceramic matrix (86% in weight), and an infiltrated polymer (14% in weight) [10]. Compositional analysis revealed that the ceramic network consists of leucite as major phase and zirconia as minor phase [11]. The polymer phase is composed of a mixture of urethane dimethacrylate and triethylene glycol dimethacrylate resins [10], and includes large amounts of carbon [12]. The manufacturing process of PICN material includes two steps: first, a porous presintered feldspar ceramic is produced; second, the porous ceramic network is infiltrated with a polymer. Before resin infiltration, the ceramic network is conditioned by a coupling agent and thus, the polymer network is chemically cross-linked to the ceramic network for the formation of an interpenetration-network system [10]. Coldea et al. [10] evaluated the effects of ceramic-network densities (59% to 72%) on flexural strength, strain at failure, elastic modulus, and hardness of four different PICN materials and concluded that the ratio between porous ceramic and polymer content affected aforementioned mechanical properties. It has been demonstrated that higher ceramic content reveals lower flexural strength and strain at failure, but higher elastic modulus and hardness [10]. PICN material is reported to have mechanical properties similar to enamel and dentin [13], and to reveal characterization between ceramics and highly filled resin-based composites [11]. PICN has been suggested to have reduced brittleness and hardness, along with a lower elastic modulus and improved fracture toughness [10]. Furthermore, better machinability of the material [10] in comparison to ceramics might provide accurate results in thinning areas of the restorations and, since the material does not require any firing procedures after milling, additional laboratory processes can be eliminated. These properties may provide the use of PICN material for different restorations, including hybrid abutments.

The hypothesis of the present study was that CAD-CAM blocks of PICN material might be an alternative, as opposed to LDS material, for the fabrication of hybrid abutments. To test the hypothesis, the aims of the study were to evaluate and compare (1) the flexural strengths of PICN and LDS materials, (2) microshear bond strengths (µSBS) of PICN and LDS specimens to a resin cement, (3) fracture resistances of PICN and LDS hybrid abutments, and (4) the effect of thermocycling on the tested parameters.

## 2. Materials and Methods

### 2.1. Specimen Preparation

**Figure 1:**
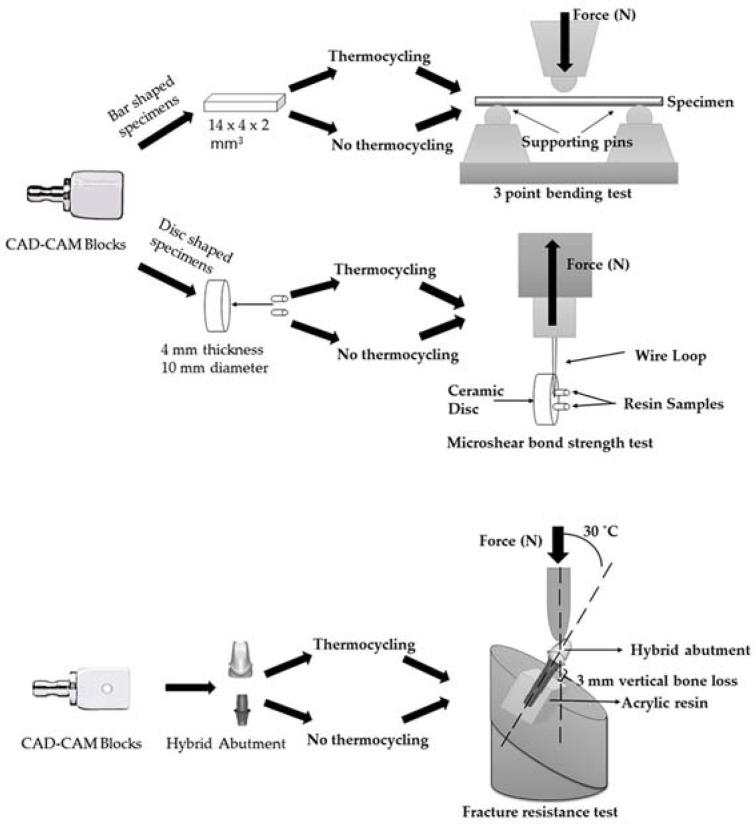
Schematic representation of specimen preparation and test designs. LDS: lithium disilicate ceramic; PICN: polymer-infiltrated ceramic-network; CAD: computer-aided design.

LDS ceramic (IPS e.max CAD, Ivoclar Vivadent, Schaan, Liechtenstein) and PICN material (Vita Enamic, Vita Zahnfabrik, Bad Säckingen, Germany) were investigated and the blocks used in this study are presented in Table 1. All specimens were milled with a five-axes CAD-CAM machine (Sirona inLab MC X5, Dentsply Sirona, PA, USA) and the specimens were fabricated in different geometries according to the test applied. A schematic illustration of specimen preparation and study design is presented in Figure 1.

For 3-point-bending and µSBS tests, 20 bar-shaped (1.2 mm × 4 mm × 14 mm) and 20 disc-shaped (4 mm in thickness and 10 mm in diameter) specimens were milled from each material, respectively. LDS specimens underwent crystallization firing at a temperature of 840 °C for 25 min in a Programat EP5000 (Ivoclar Vivadent, Schaan, Liechtenstein) furnace according to the manufacturer’s instructions.

Resin samples were prepared on the disc-shaped specimens for the µSBS test. Before the application of resin cement, the surfaces of the LDS and PICN specimens were conditioned with 5% HFA gel (IPS Ceramic Etching Gel, Ivoclar Vivadent, Schaan, Liechtenstein) for 20 and 60 s, respectively. Specimens were rinsed with distilled water for 2 min and air-dried after etching. A thin coat of Monobond Plus (Ivoclar Vivadent, Schaan, Liechtenstein) was applied to the etched surfaces with the use of a microbrush and left to react for 60 s. Two tygon tubes with a thickness of 2 mm and diameter of 1 mm were placed on each specimen. A resin luting agent (Multilink Hybrid Abutment; Ivoclar Vivadent, Schaan, Liechtenstein) was applied from a mixing syringe into the tygon tubes, and cylinder samples were left for autopolymerization for 10 min. Specimens with resin cylinders were stored in distilled water at 37 °C for 24 h. Tygon tubes were carefully removed using a sharp scalpel. The resin samples that failed before the test procedure were not included in the analysis, and new samples were prepared. Totally, 40 resin samples were obtained for each material.

**Figure 2:**
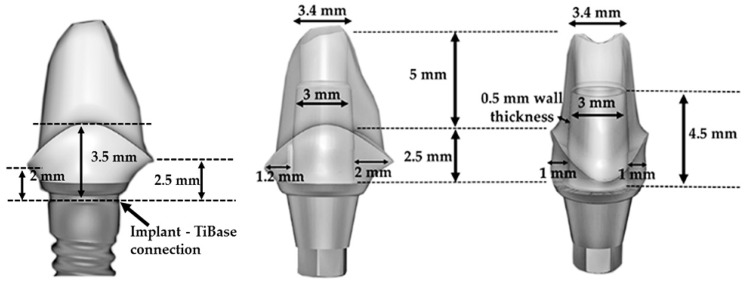
Interproximal (left and middle) and palatinal (right) views of designed custom abutment in CAD software. Dimensions of titanium insert base and ceramic coping are presented.

For the fracture-resistance test, 20 hybrid abutments from each material (totally 40) were designed and milled from the blocks for the abutments (Table 1). Before designing the custom abutment on the CAD software, to simulate the position of an implant at anterior region, an implant (Astra-Tech OsseoSpeed TX 4.5/5.0; Astra Tech, Dentsply Implants, Mölndal, Sweden) was embedded in the right maxillary central-incisor region of a phantom model (AG-3 WOK, Frasaco GmbH, Tettnang, Germany) (Figure 2). A titanium insert base (TiBase, AT OS 4.5/5.0 L; Sirona Dental Systems GmbH, Bensheim, Germany) was screwed in the implant and a scan body was placed on the titanium insert base. Digital impressions with an intraoral scanner (CEREC Omnicam, Sirona Dental Systems, Bensheim, Germany) were made for maxillary and mandibular arches and data were transferred to the CAD software. The custom abutment was designed by taking the emergence profile, adjacent teeth, and opposite arch into the consideration. Figure 2 represents the dimensions of the custom abutment. All abutment specimens of both materials were milled according to the same abutment design. LDS specimens underwent crystallization firing according to the manufacturer’s instructions. Custom abutments were cemented on the titanium insert bases using a self-curing resin luting agent (Multilink Hybrid Abutment, Ivoclar Vivadent, Schaan, Liechtenstein) and following the manufacturer’s instructions. Bonding surfaces of the titanium bases were sandblasted with 50 µm Al_2_O_3_ (Korox 110, Bego, Bremen, Germany) for 15 s at 2-bar pressure from a distance of 10 mm. A primer (Monobond Plus, Ivoclar Vivadent, Schaan, Liechtenstein) was applied on the sandblasted surface of titanium insert base and allowed to react for 60 s. The inner surfaces of the LDS and PICN abutment specimens were conditioned as described above. The luting agent was directly applied from the mixing syringe to the bonding surfaces of the titanium insert base and ceramic, parts that were tightly pressed together for 5 s. Hybrid abutments were left for 3 min to allow for the autopolymerization of the luting agent, then excess cement was removed using a lecron carver. Glycerin gel (Liquid Strip, Ivoclar Vivadent, Schaan, Liechtenstein) was applied on the cementation joint to prevent the formation of an inhibition layer and was left for 7 min, according to the manufacturer’s instructions. After the autopolymerization of the resin luting cement, the glycerin gel was rinsed off with water and the cementation joint was polished with rubber polishers.

The hybrid abutments, and bar-shaped and disc-shaped specimens (with resin cylinders) of each material group, were divided into 2 subgroups (*n* = 10), thermocycled and nonthermocycled, according to whether the specimens were subjected to thermocycling or not. Specimens in the thermocycled groups were thermocycled in distilled water for 10,000 cycles that corresponds to one year of clinical function [14] in a 5–55 °C water bath with a 20 s dwell time in a thermocycler (MTE 101; MOD Dental, Esetron Smart Robotechnologies, Ankara, Turkey). Specimens in the nonthermocycled groups were stored in distilled water for 1 day.

### 2.2. Flexural-Strength Test

Flexural-strength values of the specimens were measured with a 3-point bending test following the guidelines of ISO 6872:2015 [15]. A universal test machine (EZ-test-500 N Shimadzu, Kyoto, Japan) was employed for the test, and each bar-shaped specimen was placed on a metal fixture with a 10 mm support span and a loading rod was positioned at the center of the specimen. The load was applied perpendicular to the long axis with a crosshead speed of 1 mm/min until failure. Maximum load (N) was recorded, and flexural strength (σ) was calculated using the following formula:

σ = 3F_1_L/2bh^2^ (1)

where σ: flexural strength (MPa), F_1_: fracture load, L: the span (distance between the center of the supports), b: width of the specimen (mm), and h: thickness of the specimen (mm).

### 2.3. Microshear Bond-Strength Test

The µSBS test was conducted with the universal testing machine (EZ-test-500 N Shimadzu, Kyoto, Japan). Forty disc-shaped specimens with 80 resin samples (*n =* 20) were attached to the testing device with a cyanoacrylate adhesive (Zapit, Dental Ventures of America; Corona, CA, USA). Shear force was applied with a wire (0.2 mm in diameter) that was looped around the base of cylinder samples, which were loaded at a crosshead speed of 1 mm/min until failure. The wire loop and the center of the load cell were positioned as straight as possible to ensure the correct orientation of shear forces. The load at failure was recorded in Newtons (N) and µSBS was calculated in megapascals (MPa) using the following formula:

(2) $$µ\mathrm{SBS}\;(\mathrm{MPa})\;=\;\frac{\mathrm{Failure}\;\mathrm{load}\;\mathrm{in}\;\mathrm{newton}\;\left(N\right)}{\mathrm{Surface}\;\mathrm{area}\;\mathrm{of}\;\mathrm{sample}\;\left({\mathrm{mm}}^{2}\right)}\;$$

### 2.4. Fracture-Resistance Test

Forty implants (Astra Tech OsseoSpeed TX, Mölndal, Sweden) with a diameter of 4.5 mm, a length of 11 mm, and an internal conical connection were used for the fracture-strength test. Implants were embedded in an autopolymerizing acrylic resin (Orthojet, Lang Dental, Chicago, IL, USA) according to International Organization for Standardization (ISO) 14801 recommendations [16]. A custom-made positioning device was used to standardize the position of the implants in the acrylic resin. A 3 mm vertical distance from the platform of the implants to the acrylic resin was not covered with acrylic resin to simulate bone loss [15]. The custom abutments were connected to the implants with a 25 N·cm tightening torque using the torque wrench. After 10 min, tightening was repeated to prevent screw loosening.

Fracture-resistance measurements of hybrid abutments were carried out using a computer-controlled testing device (EZ-test-500 N Shimadzu, Kyoto, Japan). A metal jig was fabricated to hold the abutment in a position for the application of the load with an angle of 30° to the long axis of the implants [5,17]. The load was applied using a steel rod with a rounded tip of 6 mm, and the tip was placed on the palatal sides of the specimens, below 1 mm, to the incisal edge of the hybrid abutment [5]. A 0.5 mm-thick foil was placed between the rod and the abutments to ensure the distribution of force during loading. Abutments were subjected to static loading with a crosshead speed of 1 mm/min until fracture occurred and fracture loads were recorded.

### 2.5. Failure-Mode Analysis

To identify the failure mode of all specimens subjected to the µSBS test, fractured surfaces of the resin samples were examined with a stereomicroscope (Leica S8 APO; Leica Microsystems GmbH, Wetzlar, Germany) at 40× magnification. Fractures were classified as follows: adhesive failure at the bonding interface with no remnants of resin cement; cohesive failure within the resin sample; mixed failure composed of adhesive and cohesive failures.

### 2.6. Statistical Analysis

Power analysis was performed to calculate to required sample size using analysis software (G*Power, Version 3.1.9.3. for Mac, University of Dusseldorf, Dusseldorf, Germany). Sample size of each group was calculated to be 8 with 80% power and 95% confidence level at α = 0.05. Ten specimens were prepared for each test groups to ensure targeted statistical power.

Flexural-strength, bond-strength, and fracture-resistance values were separately analyzed with two-way analysis of variance (ANOVA) with the type of hybrid-abutment material and storage conditions as the main factors. Posthoc comparisons were carried out with the Tukey test when significance was detected. Values of *p* < 0.05 were accepted as statistically significant.

## 3. Results

**Figure 3:**
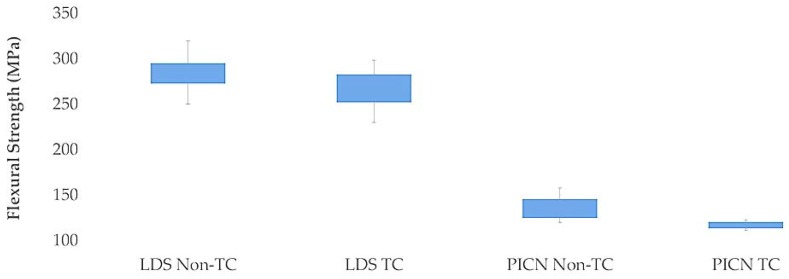
Box plot of flexural strength for each group.

**Figure 4:**
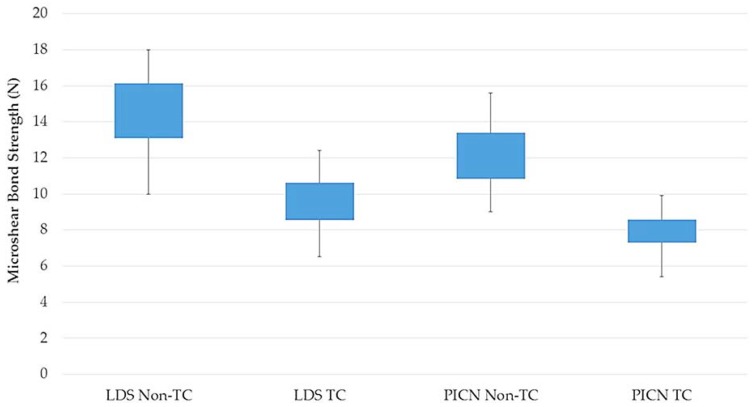
Box plot of microshear bond strength for each group.

**Figure 5:**
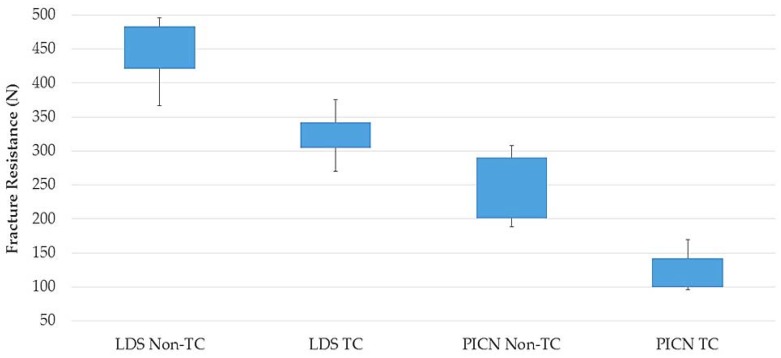
Box plot of fracture resistance for each group.

Mean flexural strength, µSBS, fracture-resistance values, and standard deviations of the thermocycled and nonthermocycled groups of each material are summarized in Table 2 and Figure 3, Figure 4 and Figure 5. Flexural-strength values revealed that both thermocycled and nonthermocycled LDS specimens showed higher flexural-strength values in comparison to PICN specimens (*p* < 0.001). A significant influence of the material factor (*p* < 0.001) on flexural strength was found. The interactions between material and storage condition were not significant (*p* = 0.579). Thermocycling did not affect the flexural-strength values of the LDS (*p* = 0.084) and PICN specimens (*p* = 0.267).

**Figure 6:**
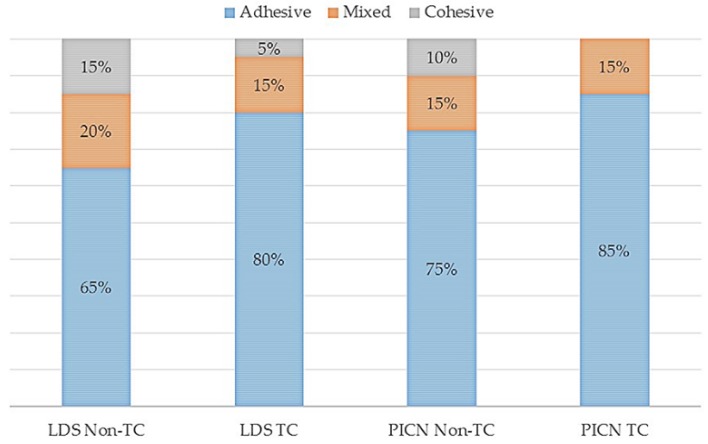
Failure modes of each group after microshear bond-strength test.

**Figure 7:**
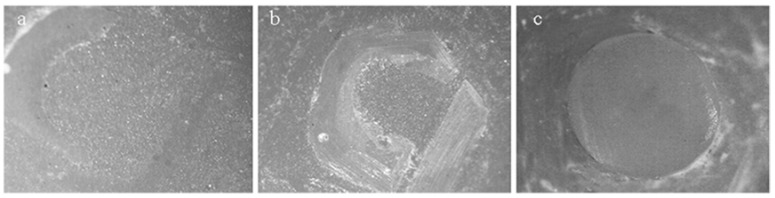
Stereomicroscope representative images showing (**a**) adhesive; (**b**) mixed; (**c**) cohesive failures of specimens subjected to microshear bond-strength test (40× magnification).

Higher µSBS values were detected between the nonthermocycled LDS material and resin luting agent in comparison to the nonthermocycled PICN material (*p* = 0.002). However, no significant difference was observed between the µSBS values of LDS and PICN specimens and resin luting agent when the specimens were subjected to thermocycling (*p* = 0.06) (Table 2). The interactions between both parameters were not significant (*p* = 0.423). The distribution of failure modes of the resin–ceramic specimens is presented in Figure 6. The predominant failure modes were adhesive failures for all groups, and frequencies of adhesive failures were higher in thermocycled groups. All groups showed mixed and cohesive failures except for the thermocycled PICN group in which cohesive failure was not observed. Representative failure types are shown in Figure 7.

**Figure 8:**
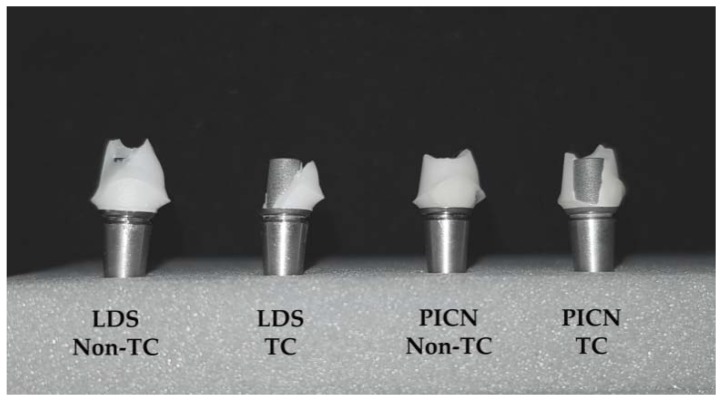
Macroscopic image showing failures of LDS and PICN hybrid abutments after fracture-resistance test. Failure within ceramic material was detected at nonthermocycled LDS and PICN abutments and ceramic detachment was observed at thermocycled hybrid abutments (LDS TC and PICN TC).

The fracture-resistance values of LDS hybrid abutments were significantly higher than PICN abutments regardless of storage condition (*p* < 0.001). When thermocycled and nonthermocycled specimens were compared within each material group, thermocycling was found to significantly decrease the fracture resistance of the abutments (*p* < 0.001). For the fracture-resistance test, there was no interaction between storage condition and the materials (*p* = 0.6918). Failures were detected on the ceramic material, and abutment screw failures were not detected. Fractures were observed to be located in the interproximal area where compressive and tensile stresses met and where ceramic copings were thinnest. Macroscopic images revealed (Figure 8) the detachment of ceramic copings from the titanium insert bases in thermocycled abutments.

## 4. Discussion

The null hypothesis of the study that PICN material might be alternative hybrid-abutment material to LDS ceramic was rejected since PICN revealed lower flexural-strength, µSBS, and fracture-resistance values in comparison to LDS ceramic.

There are several mechanical and physical properties that affect the durability and clinical longevity of materials. In the present study, flexural strengths and fracture resistances of the LDS and PICN material were compared to provide initial characterization of the materials. According to a recent review [12], studies evaluating the three-point flexural strength of PICN materials reported strength values between 124 MPa [18] to 213.1 MPa [19]. The mean strength value of PICN obtained in the current study was 136.1 MPa, which was within the range of previously reported values [12]. PICN revealed greater flexural strength than feldspathic porcelain [20]; however, lower three-point flexural-strength values were indicated for PICN in comparison to LDS [21,22,23,24], consistently with the present study. There was no study in the literature comparing the flexural strengths of LDS and PICN specimens that were subjected to thermocycling. In the present study, thermocycling did not cause significant decrease in the flexural-strength values of the LDS and PICN specimens. This finding was in agreement with previous studies that reported that LDS [25] and PICN are not influenced by thermocycling [26,27]. Thermal cycling or storing resin-based materials in water were reported to cause polymer softening due to water penetrating the resin matrix [28]. The ceramic content and interconnected microstructure of the PICN material was considered to prevent hydrolytic degradation [26,27]; thereby, it might provide resistance to thermocycling.

Fracture resistance of hybrid abutments is directly affected by the mechanical properties of ceramic coping and bonding between titanium insert base and ceramic coping. The success of this two-piece design is dependent on the fit between the titanium and ceramic parts [6]. Therefore, the µSBS of hybrid-abutment materials to resin luting agents and the effect of thermocycling on the µSBS were investigated in the present study. The resin luting agent (Multilink Hybrid Abutment) evaluated is a self-curing resin for the cementation of ceramic structures on titanium insert bases. The nonthermocycled LDS group revealed higher bond-strength values than the PICN group. Surfaces of specimens were conditioned with hydrofluoric acid and ceramic primer as recommended by the manufacturers. The effects of acid etching on LDS and PICN materials, and the reactions between the silane primer and the etched material have been reported to be different [29]. Hydrofluoric acid etching dissolves the glassy matrix and exposes the lithium disilicate crystals, thereby forming a retentive and active surface for both micromechanical interlocking and chemical interaction [30,31]. When the PICN material is etched, both polymer and glassy matrix are dissolved, and microporosities occur on the surface [32]. Greater bond-strength values detected in the LDS ceramic may be attributed to the higher silica content of the material and better chemical bonding between lithium disilicate crystals and the silane coupling agent. Consistently, in a recent study [30], higher bond-strength values were reported for LDS ceramic, although rougher surfaces were obtained in PICN material, and authors suggested that chemical interaction between hydrophobic resin and LDS ceramic provided better bonding performance rather than the micromechanical interlocking between the rougher surfaces of PICN and resin [30]. As a limitation of the current study, surface-roughness values were not determined after surface conditioning of the specimens. This would provide a better understanding of the bonding mechanism of resin cement and tested materials, and may be evaluated in further studies. For both nonthermocycled LDS and PICN specimens, adhesive failure was the most common failure type, and mixed and cohesive failures were also detected (Figure 7). Adhesive failure is directly related to the bonding interface, but cohesive failure signifies better bonding strength as failure arises due to a flaw within the resin material rather than the bonding surface. Cohesive failures can also be explained by the high bond strength that exceeded the intrinsic strength of the resin material itself [33]. PICN specimens demonstrated higher rate of adhesive failures in accordance with µSBS values. Thermocycling has been shown to decrease the bond strength of resin luting agents to LDS ceramics [34] and PICN material [35,36], and no significant difference was found between the µSBS values of thermocycled materials. The rate of adhesive failures increased for thermocycled LDS and PICN materials, which were 80% and 85%, respectively. Significantly lower µSBS values may be explained by the water absorption of the resin luting agent that could negatively affect bond durability at the ceramic–resin interface due to the plasticization of the resin luting agent and hydrolytic degradation of the silane coupling agent [37].

LDS ceramics have high initial strength; however, the brittle characteristic of LDS ceramics was reported to cause spontaneous fracture of the materials [23]. On the other hand, it has been suggested that, although polymer-containing materials do not show high initial strength, the relatively elastic microstructure of the material may prevent the propagation of cracks that occur as a result of mechanical fatigue [24]. However, in the present study, results of the fracture-resistance test exhibited that LDS hybrid abutments had greater resistance than PICN abutments. Thermocycling significantly decreased the fracture resistance of both materials. Thermal cycling might generate tension stresses that enable the initiation of cracks within ceramic specimens, which results in catastrophic failure [38]. However, in the current study, thermocycled abutments showed lower fracture-resistance values, while flexural strengths of the bar specimens were not affected by thermocycling. Therefore, the negative effect of thermocycling observed in fracture-resistance values may be attributed to resin-cement degradation. A previous study [39] reported significant decrease in retentive strength of the resin luting agent evaluated in this study after thermomechanical aging. Thermal cycling could induce the degradation of resin cement at the titanium insert base and ceramic interface and cause debonding that may affect the degree of load transfer at the interface [40]. In accordance with this suggestion, the detachment of ceramic coping from titanium insert base was observed at thermocycled hybrid abutments.

The maximum values of occlusal forces on incisors have been reported as ranging from 90 N to 370 N [41]. For the nonthermocycled and thermocycled LDS ceramic groups, failure occurred within a load range of 366.5 N to 495.4 N, and 269.7 N to 375 N, respectively. The mean fracture-resistance value of the thermocycled LDS group and PICN groups was below human occlusal forces (Table 2). However, in the present study, hybrid abutments were subjected to a fracture-resistance test without crowns to eliminate failures that might occur on crown material and/or the crown/abutment interface, since the study focused on the resistance of hybrid-abutment material. Previous studies investigating titanium and zirconia abutments reported metal deformation of screws and/or abutments and screw fracture after fracture resistance tests [42,43,44]. In the present study, failures were detected at the ceramic material and abutment-screw failures were not observed. Kim et al. [45] indicated that screw failures occurred for loads of 650 N, which is above the fracture loads detected in the current study.

In the present study, only an internal connection implant was involved for the fracture-resistance test, which can be a limitation of the study. As another limitation, the abutments were loaded 1 mm below the incisal edge, simulating class I occlusion. However, in different occlusion types, occlusal loads are positioned in different locations that can affect force distribution and fracture loads.

There are several limitations regarding the test methods used in this study. The flexural-strength test combined tensile, compressive, and shear stresses and included elements of proportional limit and elastic-modulus measurements [46]. A three-point bending test was performed to evaluate the flexural strength of the materials. However, due to the small size of the CAD-CAM blocks, specimen sizes that were described in ISO 6872:2015 [15] were modified, and, as a result, span length was shorter and test setup was miniaturized. To overcome the limitations of block sizes, a new method for assessing flexural strength was recently introduced that requires smaller test specimens with rectangular plate geometry [46]. This test method may be conducted in further studies. A favorable test method for bond-strength evaluation has been suggested to involve high bond-strength values, homogeneous stress distribution, and adhesive failures; however, no test method has been regarded as ideal so far [47]. Microbond-strength tests have some advantages over macro tests since microbond-strength tests demonstrate higher percentage of adhesive failures, lower coefficients of variation, and it is possible to evaluate different areas of the same specimen [48,49]. Microtensile and microshear bond-strength tests are generally used to assess the bond strength between resin cement and restorative materials, and the latter method was used in this study due to the less-specimen requirement of this method and easier control of the bond test area with the use of tygon tubes [50]. It is important for the bond-strength test setup to concentrate the load onto the adhesive interface, and, in the µSBS test, loading forces are applied as close as possible to the desired test site [51]. An µSBS test has been suggested as an appropriate method for evaluating bond strength of CAD-CAM ceramics to resin cement [35]. On the other hand, microtensile and micro-pushout tests provided more reliable results in comparison to the µSBS test since specimen configuration, direction of force, and elastic moduli of the materials involved have a significant effect on µSBS values and cause greater variation of the findings [52]. A recent study [47] that conducted finite-element and failure analyses to compare microbond tests concluded the use of a micro-pushout test for uniform stress concentration and adhesive failure pattern. A micro-pushout bond-strength test may be performed in further studies to obtain more reliable results. The fracture resistance of the hybrid abutments was evaluated in this study; however, this test method has been reported to provide failure data and stress distributions different from clinical situations [53]. Therefore, considering the limitations of the test methods used, the findings of this study may be regarded as initial mechanical characterization of the materials and may not be directly relevant to clinical conditions.

In this study, only LDS and PICN material were investigated. However, zirconia ceramic showed higher load capacity and reliability among the other ceramic materials used for hybrid abutments [7]. Plastic deformation of titanium alloy was also observed after loading of zirconia abutments, implying higher fracture resistance of the material. When hybrid abutments of LDS and a resin-based composite material were compared, no significant difference was found in terms of reliability [7]. Therefore, in further studies, it would be useful to evaluate the reliability of different ceramic and ceramic-like materials, including zirconia and resin-matrix ceramics, to provide a better understanding about the selection of these materials for hybrid abutments.

## 5. Conclusions

Within the limitations of this study, it can be concluded that PICN material exhibited lower flexural strength, lower bond strength to resin luting agent, and lower fracture-resistance values in comparison to LDS ceramic. Thermocycling had significant effects on the fracture-resistance and µSBS values of both the LDS ceramic and PICN material.

Based on the results of this in vitro study, LDS showed superior bonding and mechanical behavior than PICN, and PICN may not provide sufficient resistance to occlusal forces as a hybrid-abutment material. Therefore, LDS may be a preferable material for the fabrication of custom hybrid abutments in terms of flexural strength, fracture resistance, and bond strength. Further studies are needed to evaluate the reliability of PICN as a hybrid-abutment material.

## Figures and Tables

**Table 1:** Materials and specimen geometries used in this study.

Test Method	Specimen Geometry	Material	Product	Block Size (Lot)
Flexural strength	Bar-shaped (2 × 4 × 14 mm^3^)	LDS	IPS e.max CAD for CEREC and in lab	C 14 (V22343)
		PICN	Vita Enamic for CEREC/in lab	EM-14 (43230)
Microshear bond strength	Disc-shaped (4 mm thickness × 10 mm diameter)	LDS	IPS e.max CAD for CEREC and in lab	C 14 (V22343)
		PICN	Vita Enamic for CEREC/in lab	EM-14 (43230)
Fracture resistance	Hybrid abutment	LDS	IPS e.max CAD for CEREC and in lab	A 14 (L) (U14123)
		PICN	Vita Enamic Implant Solutions for CEREC/in lab	IS-14 L (58850)

**Table 2:** Mean flexural-strength, bond-strength, and fracture-resistance values and standard deviations. Same capital superscript letters in the same row and same lowercase superscript letters in the same column indicate no significant differences within each test method (*p* > 0.05).

Test Method	Storage	LDS	PICN
Flexural strength (MPa)	Nonthermocycled	294.3 (± 44.1) ^A,a^	136.1 (± 14.5) ^B,b^
	Thermocycled	264.5 (± 26.2) ^A,a^	116.9 (± 4.3) ^B,b^
Bond strength (MPa)	Nonthermocycled	14.6 (± 3.0) ^A,a^	11.9 (± 1.8) ^B,c^
	Thermocycled	9.6 (± 2.2) ^A,b^	7.8 (± 1.1) ^A,d^
Fracture resistance (N)	Nonthermocycled	451.6 (± 47.3) ^A,a^	242 (± 50.7) ^B,c^
	Thermocycled	321.4 (± 35.4) ^A,b^	124 (± 36.6) ^B,d^
